# VitAL: Viterbi Algorithm for *de novo* Peptide Design

**DOI:** 10.1371/journal.pone.0010926

**Published:** 2010-06-02

**Authors:** E. Besray Unal, Attila Gursoy, Burak Erman

**Affiliations:** Center for Computational Biology and Bioinformatics, Koc University, Istanbul, Turkey; Johns Hopkins University, United States of America

## Abstract

**Background:**

Drug design against proteins to cure various diseases has been studied for several years. Numerous design techniques were discovered for small organic molecules for specific protein targets. The specificity, toxicity and selectivity of small molecules are hard problems to solve. The use of peptide drugs enables a partial solution to the toxicity problem. There has been a wide interest in peptide design, but the design techniques of a specific and selective peptide inhibitor against a protein target have not yet been established.

**Methodology/Principal Findings:**

A novel *de novo* peptide design approach is developed to block activities of disease related protein targets. No prior training, based on known peptides, is necessary. The method sequentially generates the peptide by docking its residues pair by pair along a chosen path on a protein. The binding site on the protein is determined via the coarse grained Gaussian Network Model. A binding path is determined. The best fitting peptide is constructed by generating all possible peptide pairs at each point along the path and determining the binding energies between these pairs and the specific location on the protein using AutoDock. The Markov based partition function for all possible choices of the peptides along the path is generated by a matrix multiplication scheme. The best fitting peptide for the given surface is obtained by a Hidden Markov model using Viterbi decoding. The suitability of the conformations of the peptides that result upon binding on the surface are included in the algorithm by considering the intrinsic Ramachandran potentials.

**Conclusions/Significance:**

The model is tested on known protein-peptide inhibitor complexes. The present algorithm predicts peptides that have better binding energies than those of the existing ones. Finally, a heptapeptide is designed for a protein that has excellent binding affinity according to AutoDock results.

## Introduction

The determination of a specific peptide sequence with affinity to a particular protein surface is a problem of high degree of complexity arising from the fact that each residue of the peptide could be chosen from a pool of twenty natural amino acids. Even for a peptide with three amino acid residues, there exist 8×10^3^ possible peptide sequences. Screening of such a large number of molecules is complicated with both experimental and computational techniques. A rational methodology for specific and selective peptide sequence prediction is required. The necessary methodology should be time-efficient and be able to design peptides for given locations on a given set of proteins. A fast and global computational tool is desired. The importance, computational and experimental difficulties and the present state of the art of finding new peptides have recently been discussed and reviewed by Petsalaki et al. [1].

The complete peptide binding problem can be visualized as a three step process: (i) finding a path on the surface of the protein which defines a suitable region for the peptide to bind, (ii) finding the appropriate peptide for this path, and (iii) improving the peptide for a more stable binding required for inhibition. In some cases, the binding surface is known, and a peptide must be designed *de novo*. In other cases, a peptide is given and the best region on the surface that gives the optimum binding conditions is searched. The method of Petsalaki et al., based on a knowledge based bioinformatics approach addressed this problem and could successfully find the binding sites for the peptides. A similar ‘indirect’ design approach has been adopted by Frenkel et al. [2], using their *de novo* molecular design computational tool Pro_Ligand. Known peptides were docked to unknown locations on given proteins by Hetenyi and Spoel [3] using AutoDock. There have been successful attempts for computational peptide design that use knowledge-based search strategies and use diverse sets of statistical descriptors, different training databases, hydrophobicity scales, motif regularities, etc. [4]. Also, automated peptide binding search techniques [5], [6], [7] from known epitopes or protein libraries have been successfully used as bioinformatics tools. There have been applications of bioinformatics computational binding tools such as the sequence moment concept, artificial neural networks, fuzzy neural networks and Hidden Markov Model for checking the suitability of inhibitory peptides for binding on MHC class II proteins [4], [8], [9], [10], [11], [12], [13], [14], [15], [16]. Along similar lines, the suitability of a ligand as a drug was tested using Bayesian neural network analysis [17]. Application of genetic algorithms to the design of peptides has been an important line of research, examples of which are: *in silico* peptide screening and application of genetic algorithm to determine inhibitory peptide against Parkinson's-disease-related protein α-Synuclein [18]; peptides as thrombin inhibitors [19], [20]; evaluation of energies for peptide binding to a user-defined protein surface patch via Genetic algorithm for p53, oligopeptidase and DNA gyrase [21]; integer linear programming [22]; design of hexapeptides against stromelysin protein by Singh et al. [23], and a peptide buildup approach together with a genetic algorithm [24], [25], [26]. We have implemented genetic algorithm and a Markov model for *de novo* heptapeptide design in our recent study [27]. Most of the methods cited in this paragraph use models that depend on structural properties of the peptides and the peptide-protein complex. The prior knowledge of the structural basis of peptide-protein binding strategies is extremely important. The recent paper by London et al. [28], that reviews these strategies is important for understanding the basis of the works cited here. Important points that should be taken into consideration in the sequential generation of ligand molecules concerning the placement of fragments on the surface are discussed by Pegg et al. [29].

Our method offers a novel procedure for *de novo* peptide design that sequentially generates the peptide by docking its residues pair by pair along a chosen path on a protein. We adopt three novel approaches in our design: (i) The first one is the determination of the binding site on a given protein which we determine using the coarse grained Gaussian Network Model (GNM). Recently, we showed that the GNM identifies the surface residues that are suitable binding sites [30], [31], [32]. Once the binding region is determined, we obtain the binding path on it by docking an arbitrarily chosen peptide using AutoDock [33]. As will be described below, this path is flexible and not very restrictive. (ii) The second novel aspect of the model is the choice of the best fitting peptide to this path. We generate all possible amino acid pairs at each point along the path, calculate the binding energies between these pairs and the specific location on the protein via AutoDock, and form the statistical weight of each pair of amino acids. Once all possible pairs are determined for the full path, we form the Markov based partition function for all possible choices of the peptides using the Ising model matrix multiplication scheme [34]. We evaluate the transition probabilities based on this partition function, and select the best peptide for the given surface employing a Hidden Markov model (HMM) using Viterbi decoding [35]. The types of amino acids are the hidden variables of the algorithm to be obtained as the solution of the problem. (iii) The third novel feature of our approach is the consideration of the suitability of the conformations of the peptides that result upon binding on the surface by including the intrinsic Ramachandran potentials of the *φ*−*ψ* angles [36]. These are the observables of the Viterbi algorithm. As in the choice of the peptides, we assume that the Ramachandran torsion angles obey Markov statistics according to which a given torsion angle depends on the choice of the preceding torsion angle.

The Viterbi algorithm is an efficient way of determining the best state solution of a hidden Markov model based on a given observation sequence [37], [38] and is being used widely in the analysis of biological data and in bioinformatics area [39]. Protein structure prediction, where proteins are represented as Markov states, utilizes the Viterbi algorithm. The methodologies are summarized by Bystroff et al. [40]. Analysis of protein, RNA or DNA sequences were also achieved by the Viterbi algorithm as illustrated by a study on gene finding from DNA sequence by Sramek et al. [41]. The prediction of the topology of all-beta membrane proteins combining Viterbi and posterior algorithms was proposed [42] as a modified Viterbi algorithm. Mirabeau et al. determined novel peptide hormones by the Viterbi algorithm by training their algorithm with known receptor protein peptide hormone interactions and testing the model on peptide sequences in databases [43]. A similar Viterbi algorithm was adopted by Sonmez et al., [44]. Noguchi et al. employed the Viterbi algorithm on 3 different studies for peptide design against MHC class II proteins. Training was achieved by non-binding and binding peptides of the target proteins and tests applied to different data-set indicated that the method is able to predict binder peptide sequences [11], [14], [16].

The plan of the paper is as follows: In the first part, we define the methods needed for the Viterbi algorithm to run properly. Firstly, we define the binding site and path selection procedure. We then explain how the selected binding-path is partitioned into n number of overlapping grids, where n is the desired peptide length. Subsequently, the docking procedure of the 20 amino acids for the first site and 400 dipeptides each of the succeeding n grids on the binding-path are explained. The calculated binding energy scores from the docking procedure are used to obtain the statistical weights for the pair wise dependent choice of amino acids. Knowing the weights, the partition function for a given sequence is calculated using a matrix multiplication scheme and the transition probabilities for each n grid are obtained. We define the various regions on a Ramachandran map as the torsion states of the residues. We then construct the probabilities of the torsion states of a pair of neighboring residues using information from the Protein Data Bank. Inasmuch as we are interested in the denatured conformations of peptides, we construct a coil library from which we obtain the probabilities of the torsion angles [45]. The bound (docked) conformations of each dipeptide on each n grid are used to obtain the torsion angle state of the dipeptide of interest which is used to define the emission probabilities. In the second part of the paper, implementation of the Viterbi algorithm to the peptide binding problem is defined. In the final part, several examples are given.

## Methods

### The Model

The model consists of five parts: (i) Determining the binding site and the sequence of residues on the surface of the target protein on which the peptide will be docked, (ii) determining the chiral carbon positions of the n residue peptide that will be interacting with the sequence of residues on the target protein surface, (iii) partitioning the path of the n points into a sequence of grids, (iv) docking, by using AutoDock, all 20 peptides to the first grid, and all 400 dipeptides to the succeeding pairs of grids and evaluating their binding affinities, (v) characterizing the *φ*−*ψ* propensities of the dipeptides. We then use the outcome of these five steps for the implementation of the Viterbi algorithm.

#### Determining the binding site and the sequence of residues on the target protein

The Gaussian network model (GNM) has been shown to predict the protein residues located at specific sites for drug binding [30], [31], [32]. We employed GNM to predict ‘binding site residues’ that play major role in peptide binding.

**Figure 1:**
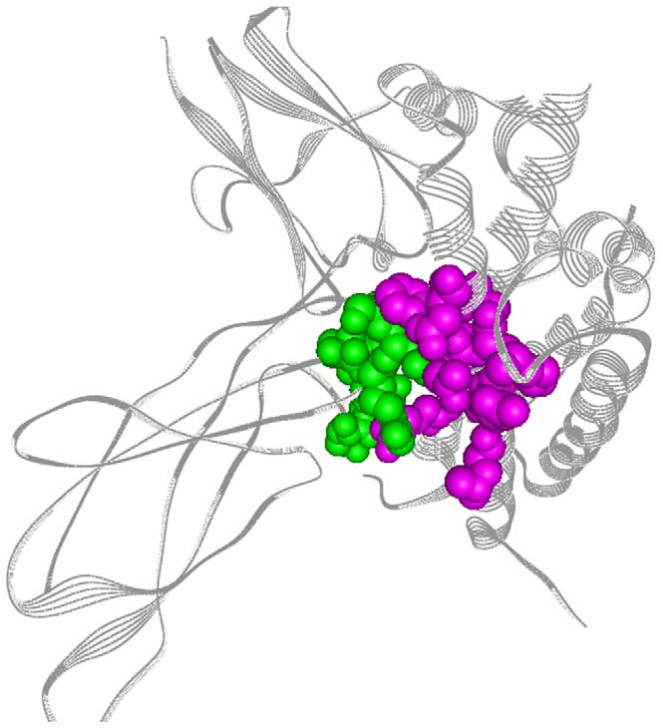
Selection of path by HotPoint server. HGHR (on the left) interacts with HGH (on the right): HotPoint predicts that the regions indicated with green and pink colors interact. The green-colored HGHR residues are selected as binding-sites. The pink-colored HGH residues are selected as binding peptide path.

Having identified a site by the GNM, we then need a sequence of residues on the protein that will be in contact with the binding peptide. We choose this sequence of residues using either of the following two approaches: (i) If the protein exists in complex with other proteins, and if the site determined by the GNM lies in an interface in the complex, then the complex is used to determine this sequence of residues on the surface. Recently, two studies indicated that protein-protein interactions, Tuncbag et al. [46] and protein-peptide interactions, Vanhee et al. [47] adopt the same structural motifs as monomeric protein folds. The existence of specific folds at the interaction site increases the stability of the formed protein-peptide complex. The counterpart fold of the target protein can be safely used as the fold of designed peptide. The protein crystal structure of the target protein is given as input to the web-server HotPoint [48] which predicts the residues of interest on the surface. In Figure 1 an example obtained from the HotPoint server is given. In this specific example the target protein Human Growth Hormone (HGH) is formed by helices and the counterpart protein Human Growth Hormone Receptor (HGHR) is formed by beta-strands. The binding site of HGH, determined by the GNM, is depicted in pink and the residues that interact with the HGH binding-site, determined by HotPoint, are in green. The region shown in pink will contain the residues to which the peptide will bind. The chiral carbons of the residues shown in green are used as grid-centers for the peptide to be designed, which will be explained in the next section. (ii) If an interacting partner of the protein does not exist, a probe peptide made up of all alanine residues, equal in number to the residues of the peptide to be designed, is docked to the binding site using AutoDock. The specific aim of docking an all alanine peptide is to obtain a path which may well be approximated by the backbone contour. Deviations from this path, due to the forces imposed by placing bulky side groups as the peptide grows on the surface for example, are accounted for as described below. The details of AutoDock parameters are given in Appendix S1. Chiral carbon coordinates of the docked peptide are essential for further steps.

#### Partitioning the Path into Grids

Our aim is to design a peptide of n amino acids. For this purpose, we need n points –one for the center of each amino acid of the peptide- that is close to the sequence of residues on the binding surface. We choose these n points as the spatial coordinates of the chiral carbons of either the docked probe peptide or the interacting protein portion determined by HotPoint. Once this path of n points is defined, n contiguous grid boxes are constructed, each centered around one of the n points. The first grid contains the first amino acid. The first and the second grids along the path contain the first and the second amino acids. The t^th^ and t+1^st^ grids contain the t^th^ and t+1^st^ residues. The n chiral carbon atoms of the path define the centers of the n grid boxes.

#### Docking of amino acids & dipeptides to the binding site

The AutoDock program [33] is used as the docking tool to quantify the binding affinity between the dipeptides and the selected protein surface. Python scripts are written to automate the docking, the probability calculation and the peptide sequence determination procedures. The binding affinity of a given peptide to the surface is determined via AutoDock which gives the affinity in terms of binding energy in kcal/mol.

All 20 amino acids are docked to the 1^st^ grid of the pre-defined path, such that their chiral carbon atoms are forced to coincide with the first grid center. All 400 possible dipeptides are docked to the first and second grid boxes, with the successive chiral carbons located at the grid centers. The pair wise docking of the dipeptides is continued in this way up to the last dipeptide along the path. Pair wise docking rests on the assumption of the Markov property. The amino acids and dipeptides are prepared by the HyperChem software [49]. The dipeptides have Ace-cap on their N-termini.

The grid map is determined by the ADT subprogram of AutoDock. The pre-determined spatial coordinates of the chiral carbon atoms on the path are used as the AutoDock grid box centers. The optimum grid box size is found by trial and error as 2.5 times the length of the distance between the first nitrogen and the last carbon along the backbone of the amino acids. The grids are set such that the chiral carbon atoms of the amino acid coincide with the grid box centers but with a freedom to rotate and translate in the box. This freedom, while keeping the dipeptides to be constrained to the neighborhood of the chosen grids is necessary to account for the side chain differences of the different amino acids. It also decreases the entropy penalty of constraining a peptide to a certain region. The parameters of AutoDock are given in Appendix S2.

Docking the dipeptide on a given grid pair leads to a set of 400 binding affinities, one set for each dipeptide, which are used to determine the probability of binding of each dipeptide to the protein binding site as explained in the following section.

#### Calculation of transition probabilities

The 20 types of amino acids and the peptide length n determine the number of states in our model; there exists 20n states for the problem. For the Markov model adopted here, one needs the transition probabilities, i.e., the probability of an amino acid occupying the t+1^st^ position, given the amino acid at the t^th^ location. The binding energies of dipeptides obtained by AutoDock are used as the statistical weights for determining the transition probabilities. We adopt the Rotational Isomeric States (RIS) approach of polymer physics which is well suited for this purpose [34], [50], [51]. For each grid the probabilities are calculated via the formulation given below by *Eqs. 1*, *2*, *3*, and *4*. For the 1^st^ grid 20 binding energies are available, while for the remaining grid pairs 400 binding energies are available; the corresponding statistical weight matrix is given in *Eq.1* and *2*, respectively. The RIS matrix multiplication scheme [34] is used to determine probabilities from the energies.

The statistical weight matrix 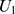 for the amino acid bound to the 1^st^ grid box is
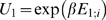
(1)where 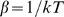, k being the Boltzmann constant and T the temperature, *i* is any of the 20 amino acids; alanine, cysteine, aspartic acid, glutamic acid, phenylalanine, glycine, histidine, isoleucine, lysine, leucine, methionine, asparagine, proline, glutamine, arginine, serine, threonine, valine, tryptophan, tyrosine.

The statistical weight matrix 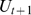 for the dipeptide formed by the t^th^ and t+1^st^ residues along the peptide is
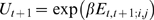
(2)where t,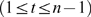, represents the amino acid position number (or the grid box number along the path). The *i*, *j* values represent any of the 20 amino acids.

The partition function, *Z*, of the peptide is obtained according to [34].
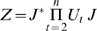
(3)where J^*^ = 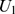 ; J = column 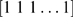. (It is to be noted that in the Flory notation, the J* matrix is given as 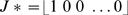 that would assign alanine to the first residue of peptide. In the present formulation, the choice of the J* matrix allows for the acknowledgement of all of the 20 amino acids to be the first residue).

The probability of having residue *i* at the t^th^ position and residue *j* at the t+1^st^ position is determined by:
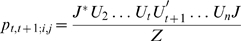
(4)Here, 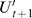 is the matrix obtained by equating all elements of 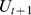 to zero except the ij^th^. The formula given above in *Eq. 4* is used to calculate the probabilities of transition states. Here, we keep the indices t and t+1, but they will be dropped in the application to the Viterbi algorithm for simplicity of representation with the understanding that each pair of sites has its own 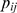.

#### Determining the emission probabilities of the φ−ψ torsion angles

A fundamental requisite for favorable binding of a peptide to the surface is that the torsion angles of the peptide in the bound conformation should not be forced to have energetically unfavorable *φ*−*ψ* torsion angle values. The apriori probabilities of the torsion angles are needed for this purpose. In this section we explain the formation of these probabilities, called the emission probabilities, and their incorporation into the Viterbi algorithm as the observable variables.

**Figure 2:**
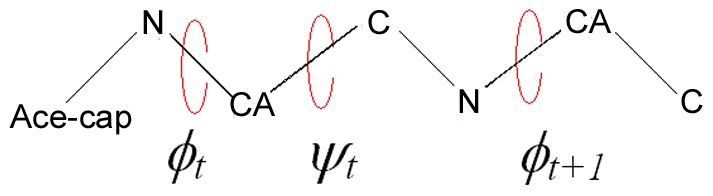
Schematic representation of torsion angles of a dipeptide. Only the backbone atoms of dipeptide are given and the acetyl cap on the N-terminal is shown as ACE, for simplicity.

**Figure 3:**
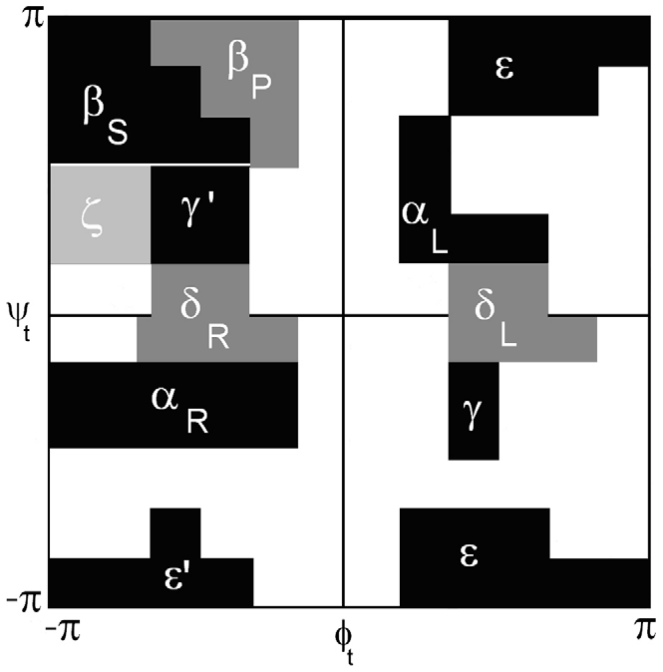
The representation of eleven states on Ramachandran map.

**Figure 4:**
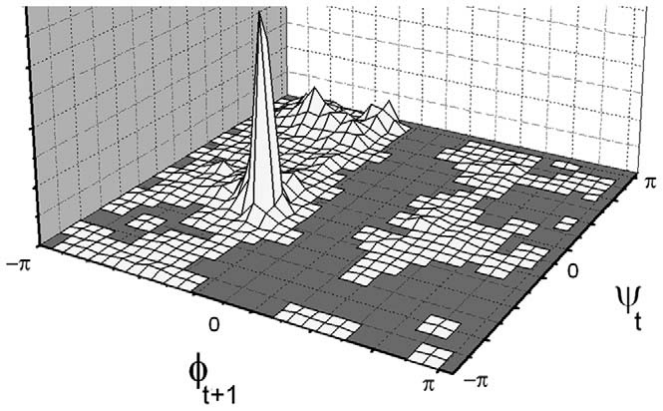
The probability distribution of 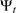
−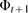 angles on Ramachandran map.

Two sets of probabilities are needed for specifying the conformation of the peptide. The first set gives the probabilities of the torsion states determined by the angles *φ_t_−ψ_t_* of the residue at the t^th^ grid. The second set gives the probabilities for the torsion angles *ψ_t_*−*φ_t+1_* of the dipeptide formed by residues at t^th^ and t+1^st^ grids. The representation of the defined torsion angles are shown in Figure 2. The *φ_t_−ψ_t_* torsion angles of the residue at grid t can select any of eleven regions, as explained below and shown in Figure 3. The torsion angles *ψ_t_*−*φ_t+1_* of the succeeding residue can choose the eleven torsion angles as explained below and shown in Figure 4. Each dipeptide is capped at its N-terminus by an acetyl group in order to define the *φ_t_* angle.

The *φ* and *ψ* angles of a residue cannot adopt all values due to backbone intrinsic torsion propensities and attractive and repulsive interactions of atoms that are in close proximity for certain combinations of these two angles. Among the repulsive interactions, steric hindrances resulting from the side groups are the most pronounced. Hydrogen bonds are the most pronounced favorable interactions. Depending on the type of the residue, these angles show preferences for different regions on the Ramachandran map. The frequency of occurrence of these regions for the twenty amino acids can be obtained from the Protein Data Bank, PDB. An examination of the frequency of occurrence of the regions for neighboring units shows that there is strong dependence on residue type [50], [51].

Plotting the *φ_t_−ψ_t_* angles on a Ramachandran map from PDB data, irrespective of residue type shows that there are eleven major isomeric torsion angle states. These are shown in Figure 3. We number the regions from 1 to eleven according to the notation of Reference [52] shown in Table 1.

**Table 1:** Notation for the eleven states.

State	Notation	Desrcription
1	ε′	Mirror image of the extended region ε
2	ε	The extended regions, *ϕ*>0, *ψ*∼ −+180°
3	α_R_	Right-handed alpha helix
4	γ	Tight turn region
5	δ_R_	The right handed bridge region between two β-strands
6	δ_L_	Mirror image of the δ_R_ region
7	ζ	Region observed mostly in residues preceding PRO
8	γ′	Inverse tight turn region
9	α_L_	Mirror image of α_R_
10	β_s_	Extended beta sheet forming region
11	β_p_	Region with extended polyproline-like helices

In obtaining Figure 3, the non-redundant PDB Select database of native proteins was used. There are 197,458 points on the Ramachandran map obtained from the database. 96% of these points fell on the eleven regions shown in Figure 3. The remaining 4% of points were outside of these regions which are known to correspond to strained conformations of the residues resulting from long range perturbations. We ignored this set of 4%.

The probability of occurrence of a residue in any of the eleven regions given above depends on the type of the library used. Since we are interested in the denatured conformations of peptides, we calculated the probabilities over a coil library that we constructed. The coil library was downloaded from the website: http://www.roselab.jhu.edu/coil/. The library contains fragments selected by Dunbrack's PISCES server according to the following criteria: less than 20% sequence identity, better than 1.6 angstrom resolution, and a refinement factor of 0.25 or better [51].

**Figure 5:**
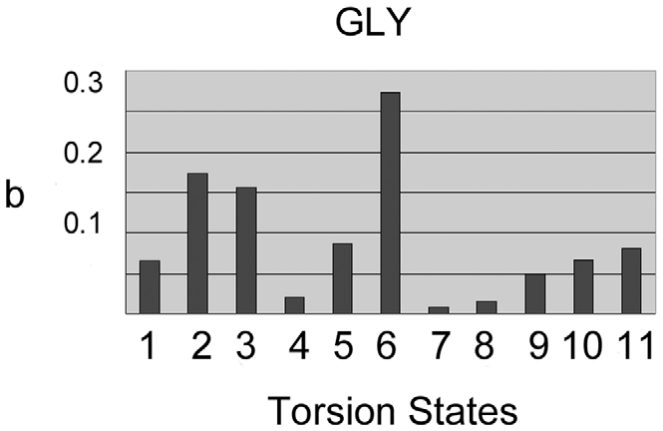
The probability distribution of GLY 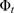
−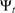 angles, derived from coil library. The dipeptide is mostly observed in the 6^th^ state; and the least observation occurs for state 7.

A given residue may be of type *i* 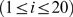 at the t^th^ grid, and may be in torsion-state *m*, which is one of the eleven regions shown in Figure 3. The frequency of occurrence of *φ_t_−ψ_t_* in torsion-states *m* for the *i* ^th^ type of residue was collected in a two dimensional array, 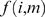 which is the normalized frequency that an amino acid is of residue type *i* in torsion-state *m*. The singlet probability 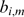 that the a residue along the peptide is of type *i* having the torsion-state *m* is
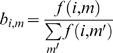
(5)For a given *i*, 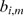 is a column vector of eleven entries. In Figure 5, 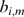 values are given for GLY as an example.

The choice of the torsion state of the t^th^ residue places restrictions on the choice of the torsion state of residue t+1 due to the dependence of the torsion angle *φ_t+1_* on *ψ_t_*. The extent of this dependence for all pairs of residues in the coil library is depicted in Figure 4 where the joint probability distribution of *ψ_t_−φ_t+1_* is presented. For uniformity of representation, we keep the same eleven regions of *ψ_t_−φ_t+1_*. In obtaining Figure 4, the non-redundant PDB Select database of native proteins was used. There are 955,679 points on the Ramachandran map obtained from the database. 91% of these points fell on the eleven regions shown in Figure 3. The remaining 9% of points were outside of these regions. We ignored this set of 9%. The probabilities are calculated over the coil library of http://www.roselab.jhu.edu/coil/.

The probability 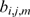 that the residue *j* is in state m of *ψ_t_−φ_t+1_* space when the preceding residue is of type *i* is
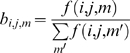
(6)For a given *ij*, 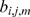 is a column vector of eleven entries.

For each n grid box, the docked conformation of the dipeptides is determined by AutoDock. Knowing the conformation leads to the *φ_t_−ψ_t_* and *ψ_t_−φ_t+1_* angles. Thus, the torsion-state of the angles is determined. Equations 5 and 6 give the apriori probability of observing the torsion-states. The product of the two probabilities, 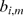 and 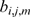, is called the emission probability for the torsion state of residue *j* when the preceding residue *i* is already prescribed. In the Viterbi algorithm below, this is indicated as 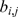. The index *m* will be removed for simplicity.

### Implementation of the Viterbi Algorithm

We follow the notation of Reference [39] in our application of the Viterbi algorithm.

We need the following definitions:

n: Number of residues of the peptide.
t: Grid number, 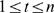

m: Index identifying the torsion state, 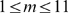

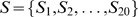
 The 20 natural amino acids set.

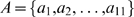
: The eleven torsion angle regions.

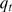
: The state of the t^th^ grid. For example 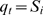 means that the state of the t^th^ grid is the amino acid *S_i_*.

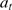
: The torsion-state of the amino acid at the t^th^ grid.

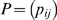
: The transition probability, 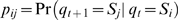.

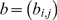
: The emission probability, 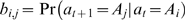

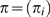
: The initial distribution vector, where 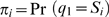.

The sets *S* and *A* describe the structure of the model, *P*, 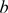 and *π* describe the parameters.

Our intention is to determine 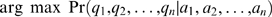, meaning a peptide sequence made up of preferable torsion angles and with high affinity to protein binding-site. The algorithm is divided into two parts. Firstly, in the forward tracking step, the algorithm finds 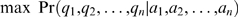. The forward tracking employs emission and transition probabilities. Then the algorithm backtracks to determine an 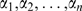 that realizes this maximum.

For an arbitrary position *t* and amino acid type *i*:

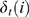
: The maximum probability of all ways to end in state 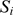 at grid *t* and have observed the torsion states 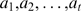.
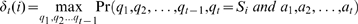
(7)where
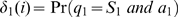
(8)then,
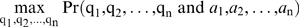
(9)is determined.

#### Initialization step



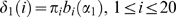
(10)

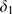
 is 1×20 array, keeping the binding affinity probabilities of each amino acid for the 1^st^ grid box. 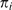 is the initial probability of binding of any 20 amino acids to the 1^st^ grid box and is a 1×20 array. All entries of 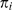 are chosen as unity to give equal chance for selection of any 20 amino acids as the initial residue of the peptide. The binding affinity and the corresponding torsion states are determined by AutoDock. The choice of the first residue will contain the information of its torsion angles from AutoDock, leading to the knowledge of the emission probability 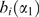 which is accounted for in *Eq. 10*.

#### Induction step



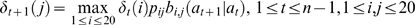
(11)

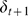
 is 1×20 array, keeping the binding affinity and torsion angle preference probabilities of each dipeptide for t+1^st^ grid.

#### Backtracking

For each grid box, the maximum probabilities are kept in 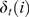 arrays, as defined in the previous section by *Eq. 11*. Backtracking of those arrays leads to the determination of a peptide sequence with a possible affinity to protein binding-site.

Initially, the last residue of the peptide sequence is determined from the 20 entries of 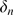, the array of the last grid box. The maximum entry of 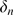 is selected, which determines the amino acid residue 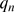.

We let
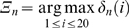
(12)and choose 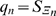. Thus, *q*_n_ is the final state of the last residue of the peptide.

The remaining 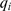, that is the amino acid types, for 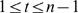 are found recursively by determining:
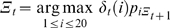
(13)and then putting 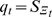. The backtracking method leads to a peptide sequence with high affinity to the target protein surface.

#### Quantifying the peptide – Target protein Interaction

The AutoDock software is used for validating the accuracy of the solutions. The tertiary structure of the designed peptide is prepared using HyperChem. The details of the procedures are given in Appendix S2. The binding affinity calculation between the target protein and the designed peptide was carried out by AutoDock.

### Test of the Algorithm

#### Protein-tripeptide case studies

As a proof of concept, the Viterbi algorithm is tested on five known protein-tripeptide complexes. The aim is to demonstrate the feasibility and the reliability of the algorithm.

The binding energy between the target protein and the peptide designed by Viterbi are determined by the AutoDock program. The inhibition constant K_i_ is also calculated by AutoDock. The visualization of complexes is achieved by Accelerys Discovery Studio 2.5 program [53].

The first complex is HIV-1 protease interacting with the tripeptide Glu-Asp-Leu. The PDB accession number of this complex is **1A30** [54]. This tripeptide is the smallest analogue of HIV-1 transframe octapeptide (TFP) Phe-Leu-Arg-Glu-Asp-Leu-Ala-Phe, which is known to be the most potent inhibitor of the target protein. The inhibition of the protein with this peptide is selective and specific.

The second complex is scytalidocarboxyl peptidase B protein interacting with the tripeptide Ala-Ile-His (**1S2K**) [55]. The protein is pepstatin-insensitive carboxyl peptidase from the organism *Scytalidium lignicolum*. The crystal structure contains the protein and the cleaved angiotensin II peptide: Ala-Ile-His. The tripeptide is bound to the catalytic residues *Gln-53* and *Glu-136* of the protein.

The other complex chosen is the signaling protein from goat mammary gland (SPG-40) and the tripeptide Trp-Pro-Trp (**1ZBW**) [56]. The protein plays role in signaling for reductive remodeling of mammary gland. The remodeling is necessary after cessation of lactation in female mammals. The protein is known to interact with oligosaccharides; the binding enhances protein-protein interactions. The tripeptide Trp-Pro-Trp sits at the active site, to which oligosaccharides bind.

The peptide deformylase (PDF) from *Enterococcus faecium* organism with Met-Ala-Ser tripeptide is another selected complex (**3G6N**), to which the peptide design algorithm is applied [57]. *E. faecium* are found in normal flora of the intestinal track, but the Vancomycin (an antibiotic) resistant types of bacteria cause infection commonly in hospitals and the strain is resistant to all commercially available antibiotics. The PDF protein is essential for bacterial growth, making it a potential drug target. The Met-Ala-Ser motif is recognized by the active site of the protein.

The last test case for tripeptides is concanavalin A (Con A) protein with Tyr-Pro-Tyr peptide (**1HQW**) [58], [59]. The peptide is determined to the best binding part out of ∼1.4×10^9^ octapeptides. A highly diverse phage library procedure is used to determine this sequence. Con A is a carbohydrate-binding protein; the literature indicates the importance of carbohydrate binding in various biological processes. Consequently, inhibition of carbohydrate-specific proteins is important for novel drug development. The chemical synthesis of oligosaccharides is a sophisticated procedure since protection of sugar monomers is complicated; peptides can be used as ligands for such protein targets. The Tyr-Pro-Tyr peptide is shown to inhibit binding of known monosaccharide ligands to Con A.

#### Protein-heptapeptide case studies

In the second step of our test, we applied the method to the design of heptapeptides against Proteinase K, and HLA-B*2705 proteins. Both of these proteins have known peptide ligands in the literature.

Proteinase K is a serine protease with broad specificity. The PDB accession number of the inhibitor peptide Pro-Ala-Pro-Phe-Ala-Ala-Ala in complex with the protein is **1P7V** from the organism *Engyodontium album*. The article about the crystal structure and interaction details of this protein-peptide complex has not yet been published.

HLA-B*2705 is a disease-associated human MHC class I allele HLA-B27 subtype protein. The protein is the target for nonapeptides. The self peptide sequence of this protein is Arg-Arg-Lys-Trp-Arg-Arg-Trp-His-Leu. The copy number of this peptide in ankylosing spondylitis patients is observed to increase [60]. Viral peptide Arg-Arg-Arg-Trp-Arg-Arg-Leu-Thr-Val derived from Epstein-Barr virus membrane has shown to have affinity to HLA-B*2705 [61]. The glucagon receptor-derived peptide Arg-Arg-Arg-Trp-His-Arg-Trp-Arg-Leu has also proven to interact with HLA-B*2705 [62]. The PDB accession codes of those 3 peptides in complex with HLA-B*2705 are **1OGT**, **1UXS**, **2A83**.

#### Predicting a peptide for a protein with no known peptide ligand

The Human Growth Hormone (HGH) is responsible for linear growth in vertebrates via stimulation of skeletal and visceral growth. The protein also plays a role in carbohydrate metabolism and fat mobilization from tissues [63]. The PDB accession code of the protein is **1HGU** [64]. A crystal structure of the protein-peptide complex is not available in PDB. Consequently, the most possible binding site of the protein is determined by GNM.

## Results

### HIV-1 protease peptide

**Figure 6:**
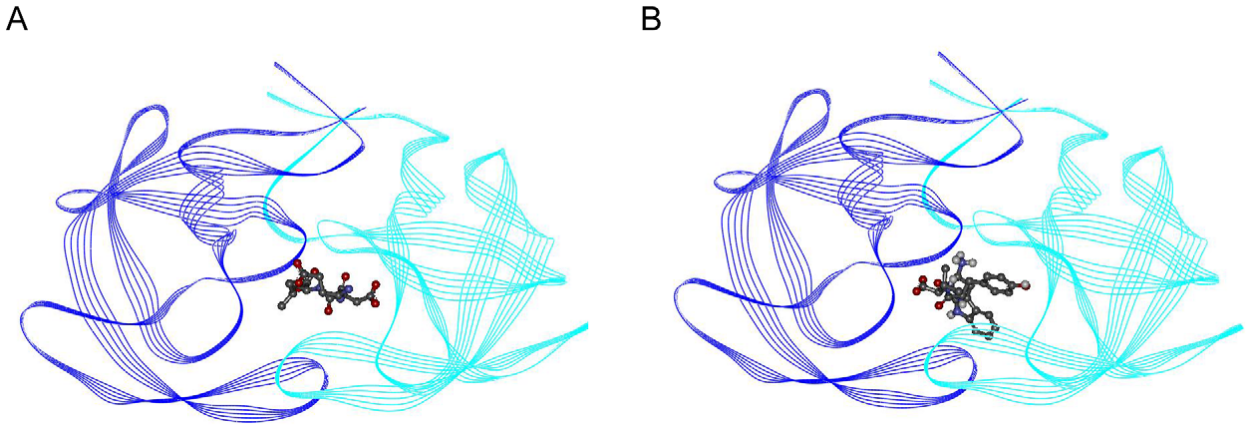
HIV-1 protease peptide complexes. (A) Glu-Asp-Leu and HIV-1 protease. (B) Trp-Tyr-Val and HIV-1 protease.

The Viterbi program designed the Trp-Tyr-Val tripeptide with high binding affinity for the HIV-1 protease. The binding affinity was calculated as −9.59 kcal/mol by AutoDock; the K_i_ value was 94.12 nanomolar. The binding affinity of the known inhibitor Glu-Asp-Leu was determined by AutoDock as −7.66 kcal/mol; the K_i_ value was 2.43 micromolar. The affinity terms are summarized in the Table 2. The binding region of the peptides is shown in Figure 6. Figure 6A indicates the complex formed by the HIV-1 protease with Glu-Asp-Leu, while Figure 6B indicates the complex formed by the HIV-1 protease with Trp-Tyr-Val. As the figures imply, both of the peptides bind to the same active-site on the protein.

**Table 2:** Binding energy; K_i_ values of HIV-1 protease peptides.

Peptide	Binding Energy (kcal/mol)	K_i_
Glu-Asp-Leu	−7.66	2.43 µM
Trp-Tyr-Val	−9.59	94.12 nM

**Figure 7:**
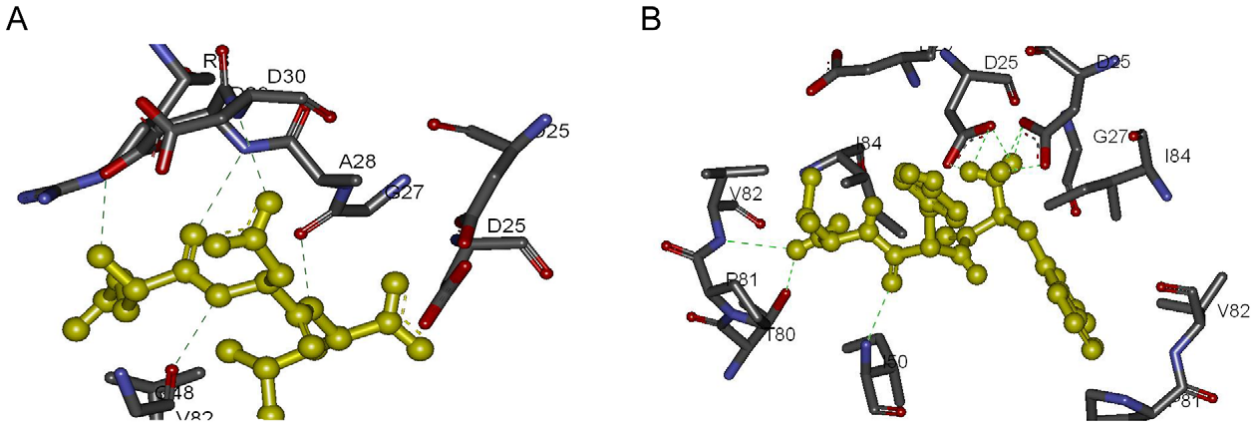
Detailed analysis of HIV-protease peptide complexes. Hydrogen bonds are indicated with green lines. (A) Glu-Asp-Leu and HIV-1 protease. (B) Trp-Tyr-Val and HIV-1 protease.

The HIV-1 protease is formed by 2 identical chains: chain A and chain B. Glu-Asp-Leu interacts with the chain A residues Asp-25, Gly-27 Ala-28, Asp-29, Asp-30, Met-46, Gly-48; and with the chain B residues Arg-8, Asp-25, Val-82. The peptide makes five Hydrogen bonds with Gly-27, Asp-29, Asp-30 and Gly-48 shown in Figure 7A. A salt bridge is present between Asp-29 of the chain A and Glu residue of the peptide. The sulfide atom of Met-46 from the chain A makes a bond with the oxygen atom of Asp.

Trp-Tyr-Val interacts with the chain A residues Asp-25, Gly-27, Ala-28, Asp-29, Asp-30, Gly-48, Pro-81, Val-82, Ile-84; and with the chain B residues Asp-25, Gly-27, Ala-28, Ile-50, Thr-80, Pro-81, Val-82, Ile-84. The peptide makes nine Hydrogen bonds with Asp-25 of the both chains, Ile-50, Thr-80 and Val-82 as shown in Figure 7B. There are 2 salt bridges between Asp-25 of the chain A and the Trp residue of the peptide. Another 2 salt bridges are present between Asp-25 of the chain B and Trp residue of the peptide.

### Scytalidocarboxyl peptidase B peptide

**Figure 8:**
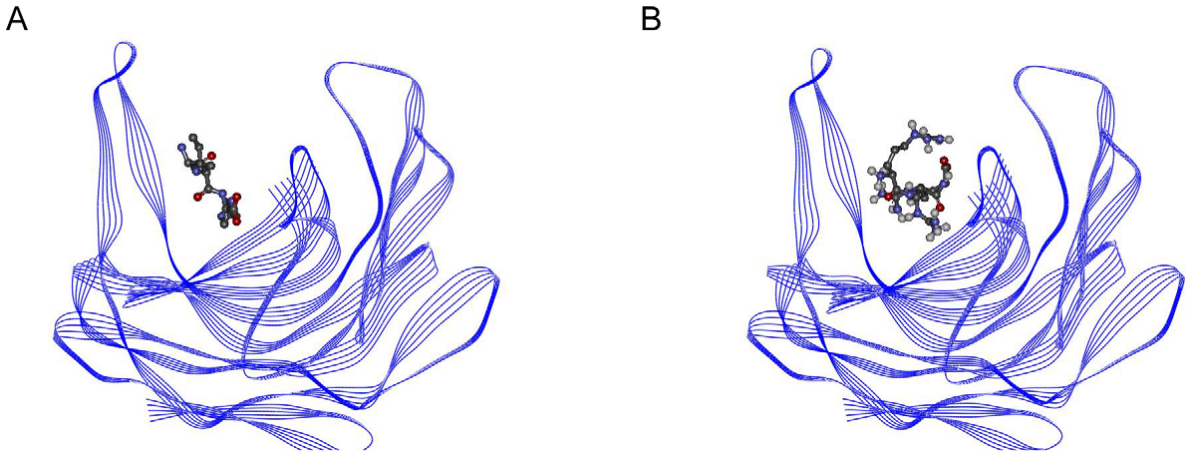
Scytalidocarboxyl peptidase B peptide complexes. (A) Ala-Ile-His and scytalidocarboxyl peptidase B. (B) Arg-Arg-Arg and scytalidocarboxyl peptidase B.

The Viterbi program designed the tripeptide Arg-Arg-Arg as potential binder peptide for the scytalidocarboxyl peptidase B protein. The binding affinity of this peptide was calculated as −12.96 kcal/mol; the K_i_ value was 315.78 picomolar. The known inhibitor Ala-Ile-His binding affinity for the target protein was determined as −5.33 kcal/mol; the K_i_ value was 124.38 micromolar. The affinity terms are summarized in Table 3. The binding region of the peptides is shown in Figure 8; Figure 8A indicates the complex formed by the scytalidocarboxyl peptidase B with Ala-Ile-His, while Figure 8B indicates the complex formed by the scytalidocarboxyl peptidase B with Arg-Arg-Arg. As the figures imply, both of the peptides bind to the same active-site.

**Table 3:** Binding energy; K_i_ values of scytalidocarboxyl peptidase B peptides.

Peptide	Binding Energy (kcal/mol)	K_i_
Ala-Ile-His	−5.33	124.38 µM
Arg-Arg-Arg	−12.96	315.78 pM

**Figure 9:**
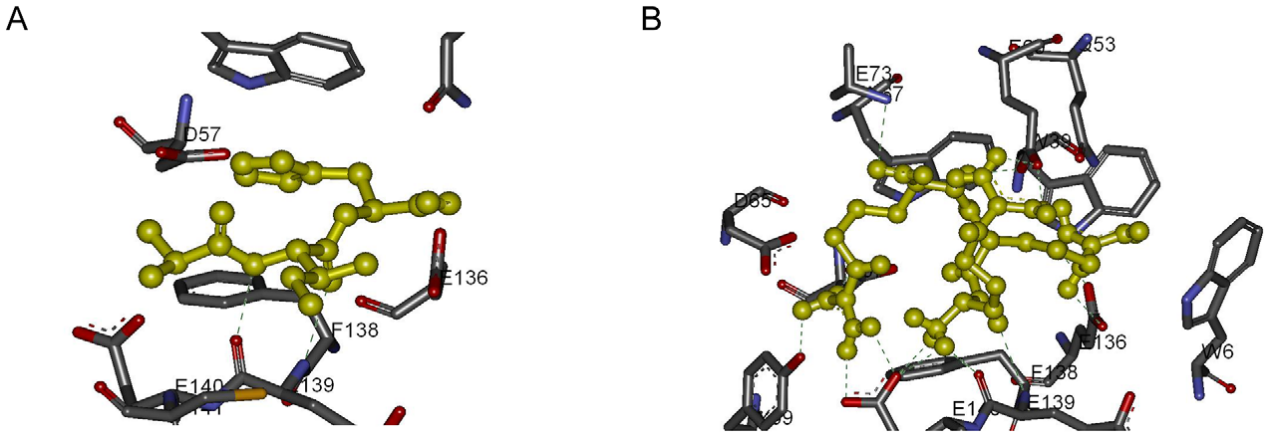
Detailed analysis of scytalidocarboxyl peptidase B peptide complexes. Hydrogen bonds are indicated with green lines. (A) Ala-Ile-His and scytalidocarboxyl peptidase B. (B) Arg-Arg-Arg and scytalidocarboxyl peptidase B.

Ala-Ile-His interacts with the residues Gln-53, Asp-57, Trp-67, Glu-136, Phe-138, Glu-139, Glu-140, Cys-141, and Cys-148. The peptide makes two Hydrogen bonds with Glu-139 as shown in Figure 9A. 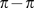 stacking between Trp-67 and His residues of the peptide is present. There is a salt bridge between Glu-139 and Ala residue of the peptide. Sulfide atom and aromatic ring interaction is present between residues Cys-141 and His; Cys-148 and His. S-O bonding is present between the residues: Cys-141 and Ala; Cys-148 and Ala.

Arg-Arg-Arg interacts with the residues Trp-6, Trp-39, Gln-53, Asp-57, Tyr-59, Asp-65, Trp-67, Glu-69, Glu-73, Glu-136, Phe-138, Glu-139, Glu-140, and Cys-141. The peptide makes fifteen Hydrogen bonds with the residues Asp-57, Tyr-59, Glu-69, Glu-73, Glu-136, Glu-139, and Glu-140 as indicated in Figure 9B. 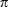
- cation interactions are observed between *P*he-138 and Arg-1 residue of the peptide; Trp-39 and Arg-3 residues. There are salt bridges Glu-69 and Arg-1; Glu-140 and Arg-1; Glu-136 and Arg-3; Asp-65 and Arg-3; also an internal salt bridge between Arg-1 and Arg-3 residues of the peptide. Arg-1 interacts with Cys-141 through S-O bonding.

### SPG-40 peptide

**Figure 10:**
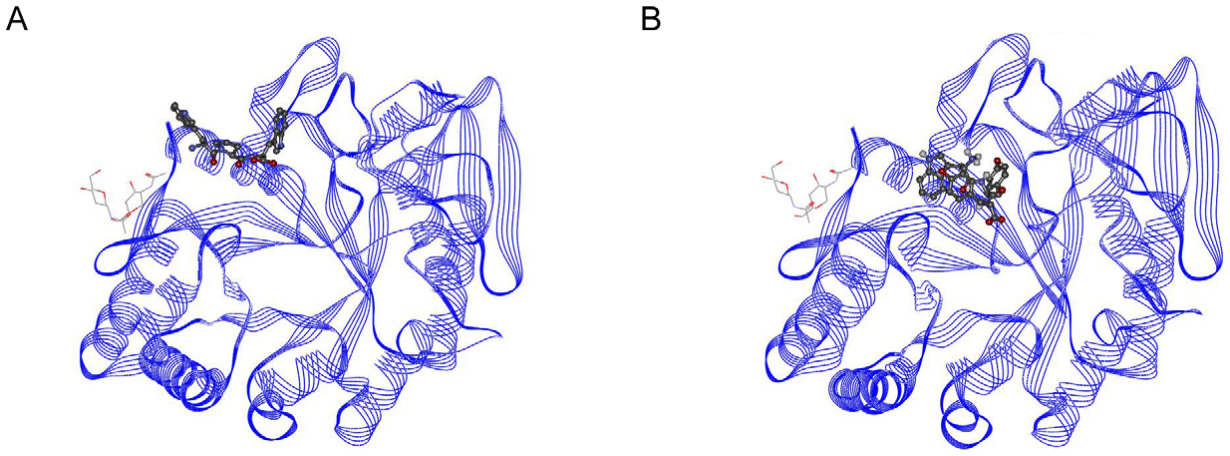
SPG-40 peptide complexes. (A) Trp-Pro-Trp and SPG-40. (B) Trp-Tyr-Tyr and SPG-40.

**Figure 11:**
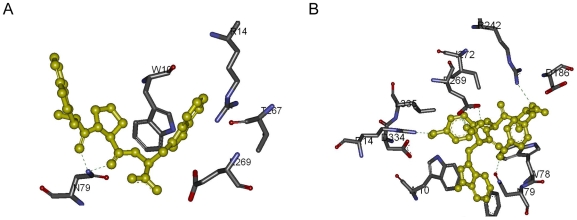
Detailed analysis of SPG-40 and peptide complexes. Hydrogen bonds are indicated with green lines. (A) Trp-Pro-Trp and SPG-40. (B) Trp-Tyr-Tyr and SPG-40.

The Viterbi program designed the tripeptide Trp-Tyr-Tyr as the sequence with possible binding affinity to SPG-40. The binding affinity of this peptide was calculated as −10.97 kcal/mol; the K_i_ value was 9.04 nanomolar. The known inhibitor Trp-Pro-Trp binding affinity for the target protein was determined by AutoDock as −9.10 kcal/mol; the K_i_ value was 215.27 nanomolar. The affinity terms are summarized in Table 4. The binding region of the peptides is shown in Figure 10. Figure 10A indicates the complex formed by SPG-40 with Trp-Pro-Trp, while Figure 10B indicates the complex formed by SPG-40 with Trp-Tyr-Tyr. As the figures imply, both of the peptides bind to the active-site of the protein. Trp-Pro-Trp interacts with the residues Trp-10, Arg-14, Asn-79, Thr-267, and Glu-269. The peptide makes two Hydrogen bonds with *Asn-79* shown in Figure 11A. There is a salt bridge between Glu-139 and Ala residue of the peptide.

**Table 4:** Binding energy; K_i_ values of SPG-40 peptides.

Peptide	Binding Energy (kcal/mol)	K_i_
Trp-Pro-Trp	−9.10	215.27 nM
Trp-Tyr-Tyr	−10.97	9.04 nM

Trp-Tyr-Tyr interacts with the residues Trp-10, Arg-14, Cys-20, Phe-37, Trp-78, Asn-79, Asp-186, Arg-242, Glu-269, Ile-272, Trp-331, Asp-334, and Leu-335. The peptide makes six Hydrogen bonds with the residues Arg-14, Arg-242, and Glu-269 shown in Figure 11B. 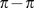 stacking is observed between Trp-10 and Trp-1 residue of the peptide and Phe-37 and Trp-1 residues. 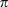
- cation interactions are observed between Trp-78 and Tyr-2; Trp-10 and Trp-1; also between Tyr-2 and Trp-1 residues of the peptides; Tyr-2 and Tyr-3 residues of the peptides. There are salt bridges between Glu-269 and Trp-1. There exists aromatic ring and sulfide interaction between Cys-20 and Tyr-2.

### PDF peptide

**Figure 12:**
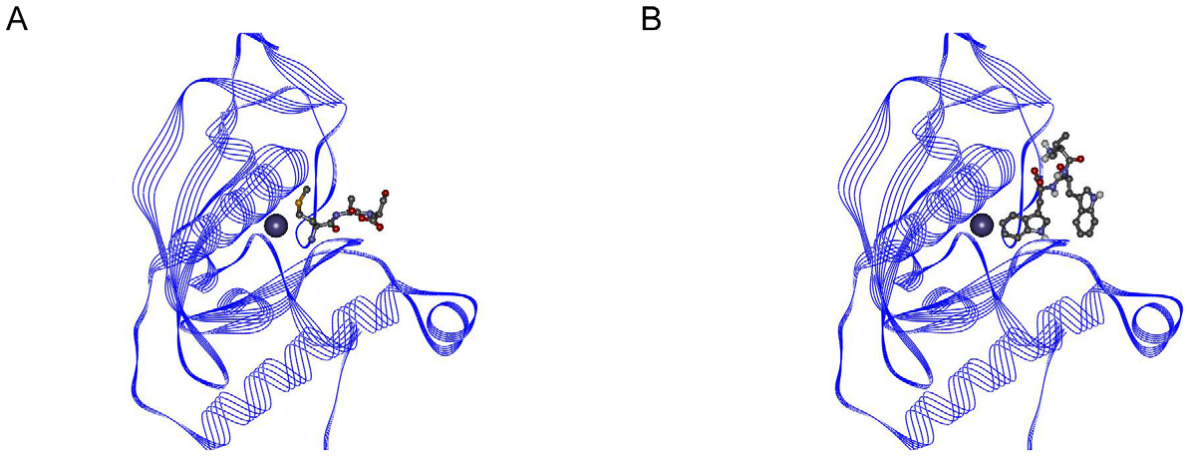
PDF peptide complexes. (A) Met-Ala-Ser and PDF. (B) Val-Trp-Trp and PDF.

The Viterbi program designed Val-Trp-Trp as peptide with possible binding affinity to PDF. The binding affinity of this peptide was calculated as −9.52 kcal/mol and the K_i_ value was 105.71 nanomolar. The known inhibitor Met-Ala-Ser binding affinity for the target protein was determined as −8.07 kcal/mol. The K_i_ value was 1.21 micromolar. The affinity terms are summarized in *Table 5*. The binding region of the peptides is shown in Figure 12. Figure 12A indicates the complex formed by the PDF with Met-Ala-Ser, while Figure 12B indicates the complex formed by the PDF with Val-Trp-Trp. As the figures imply, both peptides bind to the active-site on the protein. The Fe^+2^ ion in the active site is shown with CPK representation.

**Table 5:** Binding energy; K_i_ values of PDF peptides.

Peptide	Binding Energy (kcal/mol)	K_i_
Met-Ala-Ser	−8.07	1.21 µM
Val-Trp-Trp	−9.52	105.71 nM

**Figure 13:**
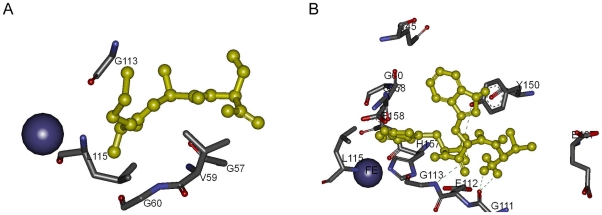
Detailed analysis of PDF and peptide complexes. Hydrogen bonds are indicated with green lines. (A) Met-Ala-Ser and PDF. (B) Val-Trp-Trp and PDF.

Met-Ala-Ser interacts with the residues Gly-57, Val-59, Gly-60, His-76, Gly-113, Leu-115, Tyr-150, His-157, His-161, and Phe-167. The peptide makes no Hydrogen bonds with the protein, Figure 13A. A sulfide atom and aromatic ring interaction occurs between the residues His-76 and Met; Tyr-150 and Met; His-157 and Met; His-161 and Met; Phe-167 and Met.

Val-Trp-Trp interacts with the residues Met-4, Gln-45, Gly-57, Gly-58, Gly-60, Leu-108, Glu-110, Gly-111, Glu-112, Gly-113, Cys-114, Leu-115, Tyr-150, His-157, Glu-158, Met-166, and Glu-187. The peptide makes four Hydrogen bonds with Gly-111, Gly-113 and Tyr-150 as shown in Figure 13B. There are internal salt bridges between Val-1 and Trp-3 residues of the peptide. There is an aromatic ring and sulfide atom interaction between the residue pairs Cys-114 and Trp-3, Met-4 and Trp-3, Met-166 and Trp-3.

### Con A peptide

**Figure 14:**
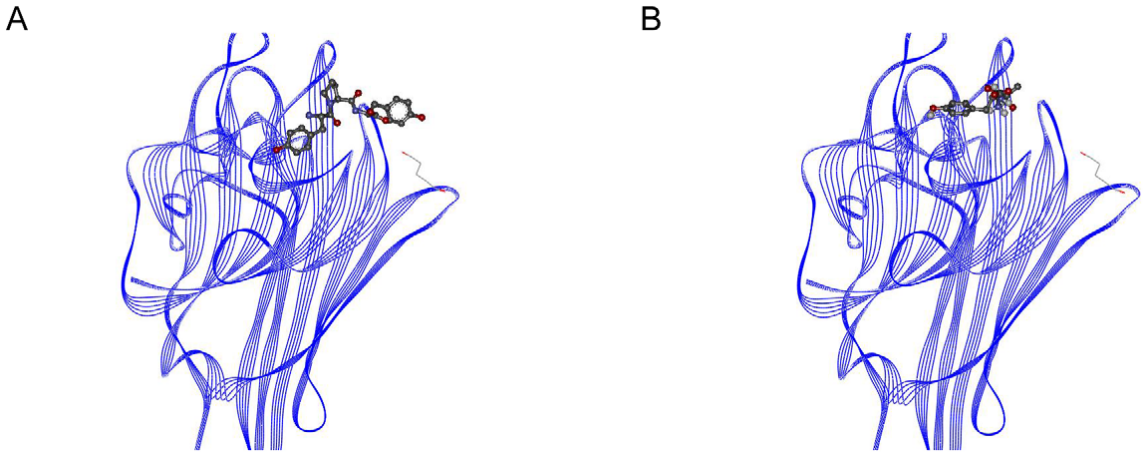
Con A peptide complexes. (A) Tyr-Pro-Tyr and Con A. (B) Gly-Ala-Tyr and Con A.

The Viterbi program designed the tripeptide Gly-Ala-Tyr for Con A. The binding affinity of this peptide was calculated as −5.70 kcal/mol; the K_i_ value was 65.83 micromolar. The known inhibitor Tyr-Pro-Tyr binding affinity for the target protein was determined as −5.70 kcal/mol. The K_i_ value was 144.38 micromolar. The affinity terms are summarized in *Table 6*. The binding region of the peptides is shown in *Figure 14*; *Figure 14a* indicates the complex formed by the Con A with Tyr-Pro-Tyr, while *Figure 14b* indicates the complex formed by the Con A with Gly-Ala-Tyr. As the figures imply, both peptides bind to the active-site of the protein. Tyr-Pro-Tyr interacts with the residues Thr-15, Ser-21. The peptide makes one Hydrogen bond with Thr-15.

**Table 6:** Binding energy; K_i_ values of Con A peptides.

Peptide	Binding Energy (kcal/mol)	K_i_
Tyr-Pro-Tyr	−5.24	144.38 µM
Gly-Ala-Tyr	−5.70	65.83 µM

Gly-Ala-Tyr interacts with the residues Thr-11, Tyr-12, Pro-13, Thr-15, Asp-16, His-205, Pro-206, and Arg-228. The peptide makes seven Hydrogen bonds with Thr-11, Tyr-12, Asp-16, Pro-206 and Arg-228. 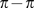 stacking is probable between Tyr-12 and Tyr residues.

### Proteinase K peptide

The Viterbi program designed the Trp-Tyr-Tyr-Tyr-Tyr-Tyr-Tyr heptapeptide with possible binding affinity to Proteinase K. The binding affinity of this peptide was calculated as −11.59 kcal/mol. The K_i_ value was 3.22 nanomolar. The known inhibitor Pro-Ala-Pro-Phe-Ala-Ala-Ala binding affinity for the target protein was determined as −8.52 kcal/mol; the K_i_ value was 569.86 nanomolar. The affinity terms are summarized in Table 7. Both peptides bind to the active-site on the protein.

**Table 7:** Binding energy; K_i_ values of Proteinase K peptides.

Peptide	Binding Energy (kcal/mol)	K_i_
Pro-Ala-Pro-Phe-Ala-Ala-Ala	−8.52	569.86 nM
Trp-Tyr-Tyr-Tyr-Tyr-Tyr-Tyr	−11.59	3.22 nM

Pro-Ala-Pro-Phe-Ala-Ala-Ala interacts with the residues Asn-67, His-69, Asn-99, Gly-100, Tyr-104, Leu-133, Gly-134, Gly-135, Gly-136, Ala-158, Gly-160, Asn-161, Asn-162, Trp-212, Ile-220, Ser-221, Thr-223, Ser-224, and Met-225. The peptide makes one Hydrogen bond with Gly-102. There exist a pi-cation interaction between Tyr-104 and Pro-1. A sulfide atom and aromatic ring interaction is observed between Cys-73, Met-225 residues and Phe-4. The sulfide atom oxygen interaction is present between Cys-73 and Ala-7. The stacking of ring structures is observed for His-69 and Phe-4.

Trp-Tyr-Tyr-Tyr-Tyr-Tyr-Tyr interacts with the residues Asn-67, His-69, Asn-99, Ser-101, Gly-102, Gln-103, Tyr-104, Leu-133, Gly-134, Ala-158, Gly-160, Asn-161, Asn-162, Tyr-169, Ser-170, Ala-172, Trp-212, Ile-220, and Ser-224. The peptide makes three Hydrogen bonds with Gln-103, Ser-170 and Asn-161. A sulfide atom and aromatic ring interaction is observed between Cys-73, Met-225 residues and Trp-1, Tyr-2, Tyr3 residues of the peptide. 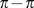 stacking is observed between His-69, Trp-212 and Trp-1; His-69 and Tyr-2; Tyr-169 and Tyr-3; Phe-192 and Tyr-4; Tyr-104 and Tyr-6; also between Tyr-5 and Tyr-6 of the peptide. A pi-cation interaction is present between His-69 and Trp-1.

### HLA-B*2705 peptide

The Viterbi program designed the Trp-Arg-Trp-Trp-Lys-Tyr-Tyr heptapeptide for HLA-B*2705. The binding affinity of this peptide was calculated as −8.97 kcal/mol; the K_i_ value was 265.82 nanomolar. The known inhibitors Lys-Trp-Arg-Arg-Trp-His-Leu, Arg-Trp-His-Arg-Trp-Arg-Leu, Arg-Trp-Arg-Arg-Leu-Thr-Val binding affinity for the target protein were determined by AutoDock as −6.84, −7.21, and −8.52 kcal/mol, respectively. The affinity terms are summarized in Table 8. Both peptides bind to the active-site on the protein.

**Table 8:** Binding energy; K_i_ values of HLA-B*2705 peptides.

Peptide	Binding Energy (kcal/mol)	K_i_
Lys-Trp-Arg-Arg-Trp-His-Leu	−6.84	9.73 µM
Arg-Trp-His-Arg-Trp-Arg-Leu	−7.21	9.73 µM
Arg-Trp-Arg-Arg-Leu-Thr-Val	−8.52	9.73 µM
Trp-Arg-Trp-Trp-Lys-Tyr-Tyr	−8.97	265.82 nM

Lys-Trp-Arg-Arg-Trp-His-Leu interacts with residues Arg-62, Ile-66, Lys-70, Thr-73, Asp-77, Tyr-99, His-114, Lys-146, Trp-147, Val-152, Gln-155, Lue-156, and Tyr-159. The peptide makes nine Hydrogen bonds with Ile-66, Lys-70, Asp-77, Tyr-84, Tyr-99, Thr-143, Trp-147, and Gln-155. There exist pi-cation interactions between Lys-146 and His-6; Lys-1 and Trp-2; Tyr-159 and Lys-1; and Tyr-99 and Lys-1. A salt-bridge is present between Asp-77 and Arg-3. A sulfide atom and aromatic ring interaction is observed between Cys-164, Met-5 residues and Trp-2. Stacking of ring structures is observed for Tyr-99 and Trp-2; Tyr-159 and Trp-2; Trp-2 and Trp- 7 in the peptide; Trp-147 and Trp-5; Trp-133 and Trp-5; Trp-147 and His-6.

Trp-Arg-Trp-Trp-Lys-Tyr-Tyr interacts with the residues Ala-69, Thr-73, Glu-76, Thr-80, Arg-83, His-114, Lys-146, Trp-147, Ala-150, Val-152, Gln-155 and Leu-156. The peptide makes three Hydrogen bonds with Thr-80, Arg-83 and Gln-155. A sulfide atom and aromatic ring interaction is observed between Cys-67 and Trp-4 residue of the peptide. 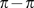 stacking is observed between Trp-147 and Trp-1; His-114 and Trp-3; Trp-147 and Trp-3; Tyr-99 and Trp-3; Trp-133 and Trp-3; Trp-147 and Tyr-6.

### HGH peptide

The Viterbi program designed Trp-Glu-Leu-Met-Phe-Phe-Tyr heptapeptide for HGH. The binding affinity of this peptide was calculated as −8.05 kcal/mol; the K_i_ value was 1.25 micromolar. The affinity terms are summarized in Table 9.

**Table 9:** Binding energy; K_i_ values of HGH peptide.

Peptide	Binding Energy (kcal/mol)	K_i_
Trp-Glu-Leu-Met-Phe-Phe-Tyr	−8.05	1.25 µM

Trp-Glu-Leu-Met-Phe-Phe-Tyr interacts with the residues Met-14, His-21, Gln-22, Phe-25, Arg-64, Glu-65, Gln-66, Thr-175, Arg-178, Cys-182, and Cys-189. The peptide makes five Hydrogen bonds with Arg-64 and Arg-178. There exist a pi-cation interaction between Arg-178 and Tyr-7. A sulfide atom and aromatic ring interaction is observed between Met-170 and Trp-1; His-18 and Met-4; Met-14, Cys-182, Cys-189 and Phe-5; Met-14, Cys-182, Cys-189 and Phe-6; Cys-182, Cys-189, Met-14 and Tyr-7. Sulfur – oxygen interactions exist between Ser-188 and Met-4; Cys-189 and Met-4; Cys-182, Cys-189 and Phe5; Cys-182, Cys-189 and Phe-6; Cys-182 and Tyr-7. Stacking of ring structures is observed for His-21 and Trp1; Phe-25 and Trp-1; His-18 and Trp-1; His-18 and Phe-5.

## Discussion

The Viterbi algorithm is successful in predicting tripeptides to the five proteins. The binding affinities of the designed tripeptides are all superior to the binding affinities of their known tripeptide ligands. The comparison is made by using the affinities given by the AutoDock. The method is also successful in predicting heptapeptides to the two proteins, proteinase K and HLA*B2705. The method was able to determine a better potential binding peptide for these two proteins.

The ability of our algorithm for *de novo* peptide design is proven for the HGH protein case-study. The binding affinity of the peptide is comparably better than some known peptide inhibitor affinities for their own target proteins.

The binding surfaces of all target proteins, except Con A, have both hydrophilic and hydrophobic residues. The Con A binding surface is made up of only hydrophilic residues. The results imply that a binding surface with both hydrophilic and hydrophobic residues could lead to a potential binder peptide design by our method. A surface exposed to water, with all hydrophilic residues, may not lead to very potent peptide designed by the Viterbi algorithm. This may be due to low number of residues in each grid box, since there is no cavity on the Con A surface. All other protein targets described in this paper have specific cavities as the binding surfaces. Consequently, the number of protein residues interacting with amino acids/dipeptides is low for the binding surfaces that are highly exposed to solvent. Balanced number of hydrophilic-hydrophobic residues in a grid box leads to more specific interactions. The specific interactions lead to design of a specific peptide with affinity to the selected protein surface.

The HIV-1 protease binding surface has −4 net charges, 50% of residues that form the surface is hydrophobic; the Trp-Tyr-Val peptide designed for this surface has two hydrophobic and one hydrophilic residues. The hydrophobic residue number is in harmony with the number of hydrophobic residues of the protein (chain A: Ile-50, Pro-81, Val-82, Ile-84; chain B: Pro-81, Val-82, Ile-84). The hydrophilic residue Tyr, which sits in the middle of the peptide, is in close proximity to the polar and basic residues of the protein surface. The known peptide of this protein has one hydrophobic and two basic residues; the only hydrophobic residue is close to Val-82 of chain B. The known peptide Glu-Asp-Leu makes interactions with only the hydrophilic and the charged residues; while the designed peptide also interacts with the hydrophobic residues of the binding pocket. Although the number of the intermolecular hydrogen bonds and the salt-bridges are the same for the protein-known peptide and the protein-designed peptide; the electrostatic compatibility between the protein and the designed peptide is more appropriate.

The Scytalidocarboxyl peptidase B binding surface has −4 net charges, 25% of residues is hydrophobic; the designed peptide Arg-Arg-Arg has +3 net charges. There are seven basic residues in the binding pocket; so the designed peptide stabilizes itself by electrostatic complementarity and the salt bridge formation. The known peptide Ala-Ile-His has two hydrophobic and one acidic residues; this peptide can interact with only 50% of the basic residues of the binding region. The designed peptide size is larger than that of the Ala-Ile-His peptide and the designed peptide also contains more positive charge. The tripeptide Arg-Arg-Arg makes a fine interaction with protein surface. The designed peptide makes three times more hydrogen bonds with the protein, when compared to the known peptide-protein interaction. The 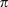 interactions and the sulfur atom bonds are observed in both the protein-known peptide and the protein-designed peptide systems. The electrostatic compatibility between the protein and the designed peptide is more appropriate.

The SPG-40 protein binding region has no net charge, since it contains one acidic and one basic residues. 20% of residues is hydrophobic; the designed Trp-Tyr-Tyr peptide has two hydrophilic and one hydrophobic residues. The only hydrophobic residue Trp is surrounded by six hydrophobic residues; four of those hydrophobic amino acids have aromatic ring in their side chains enabling the 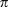 interactions. Our method designed a peptide with two Tyr residues, which are similar to saccharide monomers. Also Trp residue of the designed peptide has aromatic ring structure that is also observed in saccharides. The similarity is important since the binding molecule of the target protein is oligosaccharides. The known peptide is made up of all hydrophobic residues. The designed peptide makes more hydrogen bonds with the protein, compared to the known peptide. The 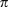 interactions and the sulfur atom bonds are observed only in the protein-designed peptide.

The peptide deformylase binding surface has no charge, 33% of residues is hydrophobic; the designed Val-Trp-Trp peptide is made up of all hydrophobic residues. The known peptide consists of two hydrophobic and one hydrophilic residues. The known peptide makes five sulfide – aromatic ring interactions; while the designed peptide makes five sulfide – aromatic ring interactions and four Hydrogen bonds.

The Con A binding surface is exposed to water with no charge; the peptide Gly-Ala-Tyr has two hydrophilic and one small hydrophobic residues. The known peptide is also made up of two hydrophilic and one hydrophobic residues. Both of the peptides have their hydrophilic residue as the 2^nd^ amino acid. The designed peptide perfectly covers the binding surface, while the known peptide is more distant to the surface. The Ala and Gly residues give flexibility to the peptide with their small side-chains. Our method kept the Tyr residue, which is similar to saccharide monomers. As stated in the case of SPG-40, the similarity to saccharides is important since the binding molecule of this target protein is also oligosaccharides.

The proteinase K binding surface has +1 net charge, 20% of residues that form the surface is hydrophobic; the 14% of residues of the designed peptide Trp-Tyr-Tyr-Tyr-Tyr-Tyr-Tyr is hydrophobic. The Asn-161 residue of protein plays role in formation of hydrogen bond with both the known and the designed peptides. The residues Cys-73, Met-225 form stable sulfide aromatic ring interactions with both the known and the designed peptides; but the number of interactions formed are higher for the Viterbi designed peptide. The aromatic ring stacking number is superior for the designed peptide, since it has more residues with side-chains containing ring structures when compared to the known peptide sequence Pro-Ala-Pro-Phe-Ala-Ala-Ala.

The HLA-B*2705 protein binding surface is made up of 44% by hydrophobic residues. Both the designed and the self-peptide have 3 hydrophobic residues, which are able to make interactions with the hydrophobic residues of binding surface. The self-peptide net charge is +3; the designed peptide charge is +2. The algorithm is successful to keep Lys and Arg residues, which are known to play major role in HLA-B*2705 binding. The residue Gln-55 is observed to play a role in hydrogen bonding for both the known and the designed peptide. The designed peptide makes three net hydrogen bonds, while the known peptide makes only two bonds.

The HGH binding region is made up of hydrophobic residues by 18%. The net charge of the surface is +2 and the net charge of the designed peptide is −1. Consequently, there exist an electrostatic complementarity between the target protein and the designed peptide. There exist two Cys and one Met residues on protein binding site, which are potential stabilizing residues of peptide binding. The designed peptide also has a Met residue, which makes interactions with the sulfur atom on its side-chain. Numerous sulfur - aromatic ring, sulfur – oxygen interactions are observed due to the Cys and Met residues of both the peptide and the protein. The Trp and Phe residues of the peptide enable stacking of aromatic rings.

The detailed analysis of the designed peptide interactions and comparison of those peptides with the known peptides indicate that our method is able to detect the requirements of binding surface; such as hydrophilicity, electrostatic compatibility, aromatic interactions, sulfur atom and its interactions.

The residues Trp, Tyr and Val are highly favored on the designed peptides. The binding energy of dipeptides containing Trp and Tyr are observed to be superior to other dipeptides. Consequently the transition probabilities of those dipeptides are favored. This may be the reason for high occurrence of those residues in the designed peptide sequences. The conservation of Trp may also be due to the formation of stacking and consequent stabilization. When the protein – designed peptide complexes are analyzed in detail, it is obvious that Trp residues form high number of stacking due to its aromatic ring; this stabilizes the formed complex. The same observation applies to Val for hydrophobic regions; since it has a smaller side-chain compared to other hydrophobic residues - except Ala -; the amino acid is able to interact with small hydrophobic cavities. The HIV-1 protease, SPG-40, HLA-B*2705 and Con A proteins have Trp and Tyr residues on their own inhibitory peptides; and proteinase K protein binding surface contains Trp, Phe and His residues on its binding surface. Nature seems to protect interactions containing amino acids with aromatic side-chains; our methodology also conserves those residues. A recent study of London et al. [28] reveals that few hot-spots on the peptide are responsible of the protein binding. The hot-spot residues are enriched in Phe, Leu, Trp, Tyr and Ile. Out of 36 amino acids of the peptides determined by VitAL, the 21 are Trp and Tyr residues. Also the peptide for the HGH protein has two Phe and one Leu residues.

Our algorithm designed a positively charged peptide for the negatively charged scytalidocarboxyl peptidase B protein surface. The method also conserved the Lys and Arg residues for HLA-B*2705 protein, which are known the play major role in peptide binding to this surface. This proves that our method not only favors the aromatic and the hydrophobic interactions, but also the electrostatic complementarity.

The only case that the method failed to determine an outstanding binder peptide is for the Con A protein case. The surface of this protein is highly exposed to water, as stated before. Consequently, we may state that our algorithm works best for cavities on protein surfaces, with both hydrophilic and hydrophobic residues present.

The study of 103 protein-peptide complexes by London et al. [28] showed that most peptides do not alter the target protein conformation minimizing the entropic cost of binding. This statement is supportive for our studies, since the protein conformation is kept rigid and the peptide is relaxed free to change conformation in AutoDock runs. London et al. also indicates that peptides of length 6–11 are observed to have coiled conformation generally. The peptides designed by VitAL are observed to have coiled conformation when bound to their target protein.

The peptide design based on a pre-determined binding surface is shown to be successful on the case-studies. Peptides are modeled as sequences of Markov chains where the states defined for each residue are dependent on the states of the neighboring residues along the chain. This assumption allows for the application of the RIS formalism to calculate the binding probability and conformational properties of the peptides. Here, we used a knowledge-based approach to determine the statistical weights of the torsion angle states of the 20 amino acids and the dependences of the statistical weights on the neighboring residues. The partition function for a given peptide is determined using the RIS multiplication of the statistical weight matrices. The Viterbi algorithm is implemented to our method in order to determine a potential binding peptide using the probability values from RIS multiplication scheme. For the Viterbi Algorithm, the binding probabilities are set as the transition state probabilities, while the torsion state probabilities are set as the emission probabilities. The peptide design; the binding affinity of the designed peptide; the peptide – protein interactions are analyzed in detail. The importance of the binding surface selection is highlighted; a binding path with no cavity and made up of all hydrophilic residues was shown to be not very suitable for determination of a candidate binder peptide. The method is shown to be successful to determine peptide according to the specific properties of the binding surface.

Haack et al. [65] indicated that the introduction of D-amino acids can significantly increase resistance to proteases and thus improve the potential use of peptides as therapeutic agents. For further improvements on VitAL we aim to add D-forms of amino acids into our library.

The algorithm requires O(mn) memory and O(mn^2^) time to run; where n is the peptide length and m is 20 - the number of states- [39]. The program details are given in Appendix S3. The time-consuming part for our methodology is the docking process. The Viterbi algorithm works efficiently to determine a single peptide is superior to other possible peptides, i.e. the most possible peptide sequence. The 1-best and posterior algorithms may also be employed to determine de novo peptide sequences, which have the same occurrence probability [42].

## Supporting Information

Appendix S1 AutoDock parameters, docking procedures.
(0.03 MB DOC)

Appendix S2 Quantifying the peptide - target protein interaction via AutoDock.
(0.02 MB DOC)

Appendix S3 Program details.
(0.02 MB DOC)
