# Supplementary material for: VitAL: Viterbi Algorithm for de novo Peptide Design
Source: PLoS One. 2010 Jun 2;5(6):e10926. doi: 10.1371/journal.pone.0010926 (PMC2880006; doi:10.1371/journal.pone.0010926)
Supplement: Appendix S1 — AutoDock parameters, docking procedures. (0.03 MB DOC) [file pone.0010926.s001.doc]

**AutoDock Parameters docking procedures**

The AutoDock program is used as the docking tool to measure the binding affinity between the amino acids / dipeptides and the selected protein surface. Python scripts are written to automate the docking; the probability calculation and the peptide sequence determination procedures. The binding affinity of a given peptide to the surface is determined via AutoDock; the program gives affinity in terms of binding energy (kcal / mol) and inhibition constant (K_i_).

*AutoDock parameters for docking the all alanine probe peptide*

The probe peptide torsion angles are set to be flexible by AUTOTORS utility of AutoDock. The polar hydrogens are added to the protein and the peptide; Gasteiger charges are added by AutoDock Tools: ADT. Genetic Algorithm option for the docking is selected. Lamarckian Genetic Algorithm is chosen as the docking search parameter. The population size is set to 150; 100 runs are made; maximum number of energy evaluations is set to 25 000 000; the number of generations is set to 50 000. The remaining parameters are set as the default values. The docked conformation of the probe peptide is then used as the path for the peptide design.

*AutoDock parameters for sequential docking of dipeptides in the chosen grids*

All 20 amino acids are docked to the 1^st^ grid of pre-defined path. All 400 possible dipeptides are docked to all remaining grid boxes on the target protein cavity. The amino acids and dipeptides are prepared by the HyperChem program; the dipeptides have an Ace-cap on their N-terminal.

The amino acid and dipeptide torsion angles are set to be flexible by AUTOTORS utility of AutoDock. The polar hydrogen and Gasteiger charges are added by ADT. The grid map is determined by ADT; the pre-determined spatial coordinates of chiral carbon atoms on the path are used as the AutoDock grid box center. The grid size is set such that the box containing the amino acid / dipeptide to be docked can rotate and translate in the box freely; with a spacing of 0.375 Angstrom between grid points. The maximum length of each amino acid and dipeptide is measured with VMD program [66]; the measured values are used for grid box size determination. Genetic Algorithm option for docking is selected. Lamarckian Genetic Algorithm is chosen as the docking search parameter. The population size is set to 150; 10 runs are done; maximum number of energy evaluations was set to 250 000; number of generations is set to 27 000. The remaining parameters are set as the default values. The docking parameters are kept small to have fast computation time; since we want to rank amino acids / dipeptides rapidly.

The docking results are analyzed and for each corresponding grid box, the amino acid / dipeptide affinity in terms of kcal/mol binding energy is determined. The calculated energy values are used to determine the probability of binding of each amino acid / dipeptide to the protein binding site as explained in the methods part. AutoDock program also gives the docked conformation of amino acids / dipeptides, which are used to determine emission probabilities for the Viterbi algorithm.
