# Supplementary material for: VitAL: Viterbi Algorithm for de novo Peptide Design
Source: PLoS One. 2010 Jun 2;5(6):e10926. doi: 10.1371/journal.pone.0010926 (PMC2880006; doi:10.1371/journal.pone.0010926)
Supplement: Appendix S2 — Quantifying the peptide - target protein interaction via AutoDock. (0.02 MB DOC) [file pone.0010926.s002.doc]

**Quantifying the peptide – Target protein Interaction via AutoDock**

The peptide is set to be flexible by AUTOTORS utility of AutoDock. Polar hydrogens are added to the protein and the peptide; Gasteiger charges are added by ADT. The grid map is determined by ADT; the pre-defined binding-site center is taken as the grid-center. An AutoDock grid with a spacing of 0.375 Angstrom between grid points is prepared. Genetic Algorithm option for docking is selected. Lamarckian Genetic Algorithm is chosen as docking search parameter. The population size is set to 150; 100 runs are done; maximum number of energy evaluations was set to 25 000 000; number of generations is set to 50 000. The remaining parameters are set as the default values.

AutoDock program gives the bound conformation of protein-peptide; the binding energy and K_i_ value. The docked conformation is used to determine the hydrogen bonds, sulfide-aromatic group interactions, disulfide bonds, sulfide-oxygen bonds, salt-bridges, and $\pi-\pi$stacking between peptide and protein residues.
