# Supplementary material for: VitAL: Viterbi Algorithm for de novo Peptide Design
Source: PLoS One. 2010 Jun 2;5(6):e10926. doi: 10.1371/journal.pone.0010926 (PMC2880006; doi:10.1371/journal.pone.0010926)
Supplement: Appendix S3 — Program details. (0.02 MB DOC) [file pone.0010926.s003.doc]

**Program details**

The Viterbi program was run on 10 core of a 32 core Linux cluster (4 nodes, 8 cores/node). For each tripeptide prediction, four hours is required to determine amino acid / dipeptide grid interactions by AutoDock; determination of transition and emission probabilities; forward and backtracking by Viterbi algorithm. The prolonged part of our methodology is docking procedure, since total of 820 docking is necessary for each program run and each docking process require three minutes to finish. Ten runs are made simultaneously, accordingly 82x3 = 246 minutes (in real time, which corresponds approximately to four hours) is essential for each tripeptide prediction. The binding affinity calculation between the target and designed tripeptide, by AutoDock, requires 10-15 hours; since very detailed docking methodology is applied.

For each heptapeptide prediction, 12 hours is required to determine amino acid / dipeptide grid interactions by AutoDock; determination of transition and emission probabilities; forward and backtracking by Viterbi algorithm. The prolonged part of our methodology is docking procedure, since total of 2420 docking is necessary for each program run and each docking process require three minutes to finish. Ten runs are made simultaneously, accordingly 242x3 = 726 minutes (in real time, which corresponds approximately to twelve hours) is essential for each heptapeptide prediction. The binding affinity calculation between the target protein and the designed heptapeptide, by AutoDock, requires 45-50 hours. Therefore, the maximum number of energy evaluations is set to 25 000 000, and the number of generations is set to 50 000. The computation time of AutoDock increases with the increasing number of energy evaluations and the number of generations.
